# Real-World Impact of Electronic Patient-Reported Outcomes on Early Intervention Among Older Patients With Lung Cancer: Prospective Cohort Study

**DOI:** 10.2196/97890

**Published:** 2026-07-06

**Authors:** Yasuhiro Nakajima, Tetsushi Maesako, Takashi Murata, Daisuke Minami, Toshiyuki Minami, Arihiko Kanehiro, Kozo Kuribayashi, Takashi Kijima

**Affiliations:** 1 Department of Respiratory Medicine, Himeji St. Mary's Hospital, 650 Nibuno, Himeji, Hyogo, 6700801, Japan, 81 792655111, 81 792655001; 2 Department of Respiratory Medicine, Hyogo Medical University Hospital, Nishinomiya, Hyogo, Japan

**Keywords:** lung cancer, electronic patient-reported outcome, ePRO, digital transformation, geriatric oncology, ecological momentary assessment, EMA

## Abstract

**Background:**

Electronic patient-reported outcomes (ePROs) have demonstrated greater sensitivity in detecting adverse events than clinician-reported outcomes. However, conventional care faces challenges related to recall bias, and evidence from real-world populations of older adults is limited. This study evaluates the feasibility of an ePRO system integrated with ecological momentary assessment and ecological momentary intervention (EMI) to facilitate a concordance model within the framework of digital transformation in health care.

**Objective:**

This study aimed to evaluate the satisfaction, usability, and clinical impact of an ePRO system integrated with ecological momentary assessment and EMI in a real-world cohort of older patients with lung cancer and to investigate its ability to facilitate a patient-centered concordance model within the framework of digital transformation in health care.

**Methods:**

In this single-center prospective cohort study, 35 patients with lung cancer used the Welby My Carte ONC app to record daily symptoms and visual data. Satisfaction and usability were evaluated using 10-point scales at the end of the first and third treatment cycles. The study focused on the clinical impact of EMI triggered by ePRO data, such as early detection of toxicity.

**Results:**

The cohort included a high proportion of older patients (20/35, 57%) aged 70 to 89 years. Satisfaction and usability scores significantly improved over time (satisfaction: 6.0 to 8.0, *P*<.001; usability: 5.0 to 8.0, *P*<.001). ePRO monitoring enabled the early detection of critical events, including immune-related uveitis (case 1), Stevens-Johnson syndrome (case 2), rare drug-induced purpura (case 3), and the monitoring of infusion-related reactions (case 4).

**Conclusions:**

ePRO monitoring is feasible for older patients with lung cancer and enhances safety through timely EMI. It serves as a core component of a comprehensive patient support program and supports the transition to a more active concordance model, ultimately improving patient well-being.

## Introduction

Patient-reported outcomes are measures of a patient’s health status that come directly from the patient, without interpretation by a clinician. In oncology, electronic patient-reported outcome (ePRO) systems have gained significant attention for their potential to enhance symptom management and clinical outcomes. Previous research, most notably the landmark study by Basch et al [1], demonstrated that ePRO systems are more sensitive and reliable in detecting adverse events, such as fatigue, nausea, and diarrhea, than clinician-reported outcomes. Furthermore, subsequent large-scale randomized trials have shown that ePRO-based symptom monitoring during routine cancer treatment can significantly improve overall survival and quality of life [2]. Recent evidence has further expanded on this by highlighting that high patient satisfaction and acceptability are critical for successful ePRO implementation, particularly among older populations who may initially perceive digital tools as burdensome. Despite these advantages, conventional outpatient care is inherently limited by recall bias, as patients are often required to retrospectively report their symptoms over several weeks during periodic clinic visits. To address this limitation, ecological momentary assessment (EMA) and ecological momentary intervention (EMI) have been proposed [3]. EMA allows real-time symptom recording in a patient’s natural environment, thereby enhancing ecological validity and eliminating recall bias, while EMI uses these data to provide immediate, personalized clinical support [3]. For older patients, who frequently face specific challenges such as cognitive decline, visual impairment, limited digital literacy, and social isolation, the real-time nature of EMA and EMI is particularly important for preventing the oversight of subtle but critical symptom changes. The integration of these digital tools is a cornerstone of digital transformation in health care. In this study, the ePRO system is positioned not merely as a stand-alone monitoring tool but as a central component of a comprehensive patient support program (PSP) [4]. This program integrates education, symptom management, and multidisciplinary collaboration to bridge the communication gap between clinic visits, similar to integrated care models that have shown benefits for patients with lung cancer [5]. In the context of lung cancer treatment, digital transformation in health care aims to move beyond passive patient compliance toward a patient-centered concordance model [6]. This model emphasizes a partnership between health care providers and patients, in which decisions are made based on shared goals and the patient’s lived experience. Such shared decision-making is further supported by the integration of outcome information into clinical conversations [7]. However, a gap remains in the evidence regarding the longitudinal feasibility and patient satisfaction associated with such systems in real-world aging populations with cancer. Therefore, this study aimed to bridge the gap between clinical trial evidence and real-world practice. By evaluating the satisfaction, usability, and clinical impact of an ePRO-EMI system in older patients with lung cancer, we investigated whether digital transformation in health care can effectively support a patient-centered concordance model and improve patient well-being in a real-world setting.

## Methods

### Study Design and Setting

This single-center prospective cohort study was conducted at Himeji St Mary’s Hospital in Himeji, Japan, between 2023 and 2024. The institution was selected as it serves as a regional core center for oncology, providing a high volume of systemic therapies for a diverse, real-world population of patients with lung cancer. The study analyzed longitudinal data obtained from routine clinical practice to bridge the gap between clinical trial evidence and real-world implementation.

### Participants and Enrollment

Eligible participants included patients with lung cancer receiving systemic therapy, including cytotoxic chemotherapy, immune checkpoint inhibitors (ICIs), and molecularly targeted therapies. Sampling was conducted on a consecutive basis for all eligible patients at our institution during the study period. As the ePRO system was implemented as part of standard care for self-monitoring, all eligible patients were encouraged to participate. The decision to use or withdraw from the app did not affect the standard of medical care provided.

### The ePRO Software and Workflow

The ePRO system used was Welby My Carte ONC (Welby Inc). The software interface allowed patients or their caregivers to record daily symptoms (eg, fatigue and appetite loss), psychological status, and physical findings through photographs (eg, skin rashes). To support adherence, particularly among older patients, the app provided push-notification reminders and visual tracking of symptom trends. Training and onboarding were provided by health care staff at the time of treatment initiation. Data were transmitted in real time to a secure dashboard accessible by the clinical team, facilitating immediate clinical decision-making through an integrated EMA-EMI framework. Representative screenshots of the Welby My Carte ONC platform are provided in Multimedia Appendix 1. A schematic overview of the EMA-EMI clinical workflow is shown in Multimedia Appendix 2.

### Measures and Clinical Data

Patient-reported outcomes were evaluated using structured questionnaires at the end of the first cycle (interim evaluation) and the third cycle (final evaluation). The questionnaire assessed four quantitative items: (1) satisfaction with the app, (2) ease of use (usability), (3) willingness to continue using the app, and (4) clarity of the instructions provided by staff. Each item was scored on a 10-point Likert scale, where 1 represented “strongly disagree” or “very poor” and 10 represented “strongly agree” or “excellent.” Open-ended responses were also collected to identify perceived advantages and challenges. Clinical covariates, including age, sex, Eastern Cooperative Oncology Group performance status (PS), disease stage, and line of therapy, were extracted from electronic medical records. Because the questionnaire was designed as a pragmatic usability assessment tool for routine clinical implementation, formal psychometric validation was not performed. The complete questionnaire items used for patient evaluation are provided in Multimedia Appendix 3.

### Clinical Impact and Case Selection

The clinical impact of the system was evaluated based on the frequency and nature of physician-initiated interventions (EMIs) triggered by ePRO data, such as telephone consultations, early clinic visits, and dose modifications. To illustrate the clinical utility of the system, 4 representative cases were selected based on their diversity of toxicity types (eg, immune-related and drug-induced) and their demonstration of how real-time monitoring can lead to improved safety.

### Statistical Analysis

Descriptive statistics were used to summarize patient characteristics and clinical data. For longitudinal comparisons of ePRO scores between cycle 1 and cycle 3, the Wilcoxon signed-rank test was used. This nonparametric approach was selected because of the ordinal nature of the 10-point scale data and the relatively small sample size. Missing questionnaire data were handled using complete case analysis. Qualitative data from open-ended responses were analyzed using a descriptive thematic analysis framework. The process involved an inductive approach in which 2 researchers independently reviewed the free-text comments to identify recurring patterns and codes. These codes were then grouped into overarching themes. Discrepancies in thematic assignment were resolved through consensus among the research team. The qualitative findings were intended to provide exploratory supplementary insights that complemented the quantitative results.

### Ethical Considerations

This study was conducted in accordance with the Declaration of Helsinki and was approved by the institutional review board of Himeji St Mary’s Hospital (S024-001). All participating patients provided written informed consent prior to data collection.

### Reporting Guidelines

This study was reported in accordance with the STROBE (Strengthening the Reporting of Observational Studies in Epidemiology) guideline for observational cohort studies. The completed STROBE checklist is provided in Multimedia Appendix 4.

## Results

### Patient Characteristics

A total of 35 patients with lung cancer were included in the analysis (Table 1).

The cohort was characterized by a high proportion of older individuals, with 20 (57.1%) of the patients aged ≥70 years (n=11 aged 70-79 years and n=9 aged 80-89 years). Most participants were male (24/35, 68.6%) and maintained a good PS (PS 0: n=23; PS 1: n=10). Treatment regimens were diverse, reflecting real-world practice: 19 patients received chemotherapy combined with ICIs, while others received cytotoxic chemotherapy (n=6), ICI monotherapy (n=4), tyrosine kinase inhibitors (TKIs; n=4), or TKI-chemotherapy combinations (n=2).

**Table 1.** Patient characteristics (N=35).

Characteristics	Patients, n (%)
Age group (years)	
40-49	1 (2.9)
50-59	5 (14.3%)
60-69	9 (25.7)
70-79	11 (31.4)
80-89	9 (25.7)
Sex	
Male	24 (68.6)
Female	11 (31.4)
Performance status	
0	23 (65.7)
1	10 (28.6)
≥2	2 (5.7)
Treatment regimens	
Cytotoxic chemotherapy	6 (17.1)
Chemotherapy+immune checkpoint inhibitor	19 (54.3)
Immune checkpoint inhibitor–based therapy	4 (11.4)
Tyrosine kinase inhibitor–based therapy	4 (11.4)
Chemotherapy+tyrosine kinase inhibitor	2 (5.7)

### Quantitative Patient-Reported Outcomes

**Figure 1.**
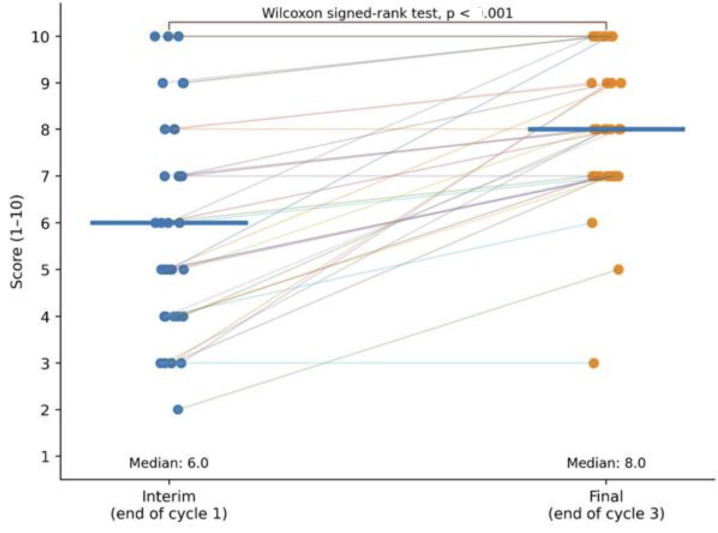
Satisfaction with the electronic patient-reported outcome app.

**Figure 2.**
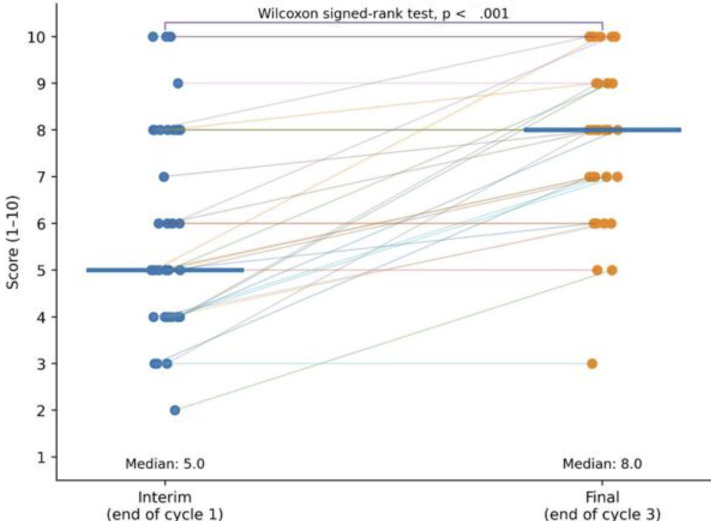
Usability of the electronic patient-reported outcome app.

**Figure 3.**
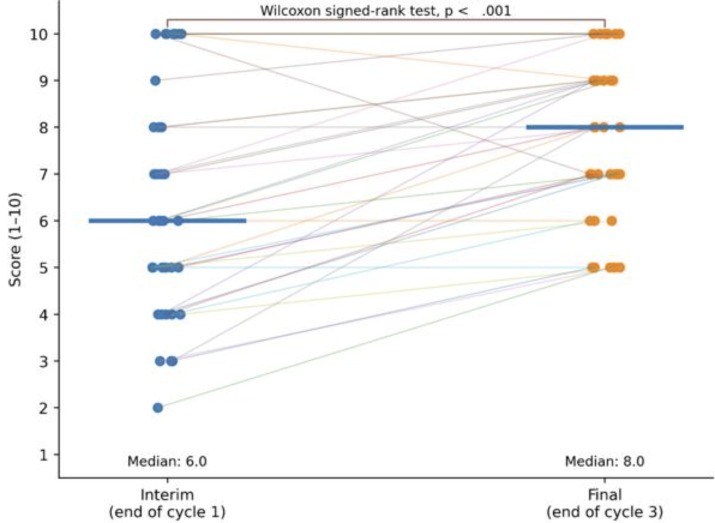
Willingness to continue using the electronic patient-reported outcome app.

**Figure 4.**
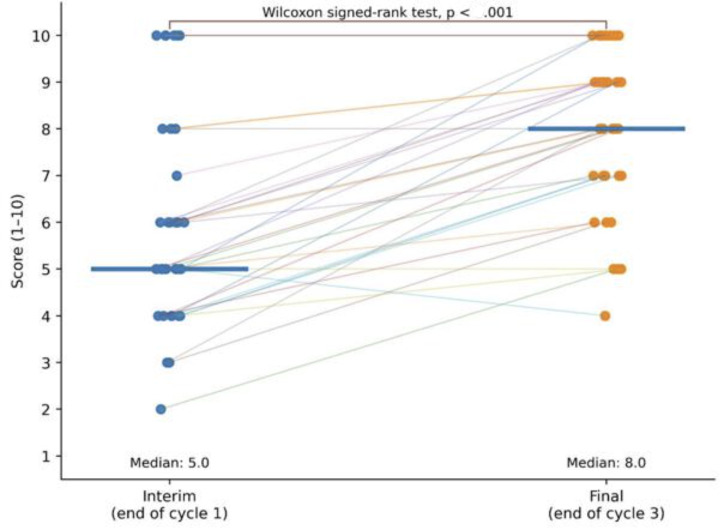
Patient-reported clarity of instructions provided by clinical staff.

Quantitative evaluations revealed that patients successfully adapted to the ePRO system over the course of 3 treatment cycles. All measured items showed significant improvement from the interim evaluation to the final evaluation. Specifically, the median patient satisfaction score increased from 6.0 at the interim evaluation (cycle 1) to 8.0 at the final evaluation (cycle 3; *P*<.001; Figure 1). Usability scores, reflecting ease of use, increased from a median of 5.0 to 8.0 (*P*<.001; Figure 2). Willingness to continue using the app improved from a median of 6.0 to 8.0 (*P*<.001; Figure 3). Furthermore, the clarity of the instructional guidance provided by the clinical staff showed a significant upward trend, with median scores increasing from 5.0 to 8.0 (*P*<.001; Figure 4). These findings suggest that even older patients can achieve high levels of proficiency and satisfaction with digital monitoring tools through repeated use.

### Qualitative Feedback: Advantages and Challenges

The thematic analysis of qualitative feedback identified 4 primary areas of focus regarding the patient experience. First, daily ePRO input was found to enhance self-monitoring and provide a significant sense of psychological security. By recording symptoms daily, patients reported that they could more effectively distinguish between expected treatment-related adverse events and general daily fatigue, thereby reducing unnecessary anxiety. Real-time connectivity with the clinical team further contributed to a perceived sense of security, particularly during the early phases of treatment. Second, patients consistently preferred the digital interface over traditional paper-based documentation. Participants highlighted the convenience and low burden of mobile data entry, as well as the clinical utility of longitudinal symptom visualizations, which enabled clearer communication of symptom trajectories during outpatient visits and reduced reliance on retrospective recall. Third, the system facilitated multigenerational support, particularly among the oldest participants. Family members frequently assisted with data entry, allowing them to become more actively involved in the care process while also providing a practical means of monitoring the patient’s condition remotely. Finally, despite these benefits, several technical and functional barriers were identified. Some patients expressed uncertainty regarding successful data transmission and suggested the inclusion of automated confirmation messages following submission. Participants also indicated that the integration of educational resources, such as a built-in reference guide to treatment-related adverse events, would enhance their ability to interpret symptom changes and manage treatment expectations. Collectively, these findings suggest that the perceived benefits of ePRO extended beyond symptom monitoring to include psychological reassurance and family engagement, while the principal challenges were related to communication feedback mechanisms and patient education.

### Clinical Impact and Representative Case Narratives

The clinical utility of the ePRO-EMI framework was demonstrated through its ability to capture clinically significant adverse events that might otherwise have remained undetected between standard outpatient visits. The system facilitated rapid physician-initiated interventions (EMI) across a variety of toxicity profiles, as illustrated by the following 4 representative cases selected to show the diversity of detected events. In the first case, a 71-year-old male reported “foggy vision” via the app on the first day of pembrolizumab treatment. This early EMA data led to a prompt hospital visit on day 9, resulting in the diagnosis and successful management of grade 2 immune-related uveitis without treatment interruption. Similarly, a 40-year-old female used the image upload function to report a skin rash. Real-time monitoring of these images allowed clinicians to detect the rapid progression of toxicity from grade 2 to grade 3, leading to an immediate diagnosis of Stevens-Johnson syndrome and life-saving corticosteroid intervention.

The system also proved effective in identifying rare toxicities, as demonstrated by the case of a 83-year-old female who uploaded photographs of lower-extremity skin lesions. This led to the diagnosis of epidermal growth factor receptor-TKI–associated purpura and enabled a flexible dose modification to maintain treatment continuity. Finally, the ePRO system provided a continuous layer of safety monitoring for a 50-year-old female who had experienced severe infusion-related reactions to amivantamab. Postdischarge monitoring confirmed the absence of symptom exacerbation, allowing safe continuation of therapy in an outpatient setting and reducing patient anxiety. These cases highlight how the integration of real-time patient data into clinical workflows can directly enhance patient safety.

## Discussion

### Principal Findings

This study demonstrated that an ePRO system integrated with the EMA-EMI framework significantly enhanced patient satisfaction and usability over time while enabling the early detection of critical adverse events in a real-world population of older adults with lung cancer. Specifically, the longitudinal improvement in median scores from 6.0 to 8.0 across satisfaction and usability metrics suggests that older patients can effectively adapt to digital health tools when provided with continuous institutional support. Our findings regarding feasibility and satisfaction align with recent evidence highlighting the potential of ePRO systems in older adults. While previous studies have often noted lower completion rates in older adult cohorts, Riedl et al [8] observed that home-based ePRO systems are feasible for >80% of older adults who survived cancer when family and staff support are integrated. The significant increase in scores for clarity of instructions (Figure 4) in our cohort suggests that the initial digital barrier can be overcome through repeated use and proactive staff engagement, leading to high levels of acceptance. The clinical impact of the ePRO-EMI system was particularly evident in the early detection of potentially life-threatening toxicities, bridging the gap between periodic outpatient visits. For instance, the detection of immune-related uveitis (case 1) within the first week of treatment is crucial, as this condition can manifest much earlier than the reported median latency of 12 weeks [9]. Similarly, real-time monitoring of image data in case 2 allowed the immediate diagnosis of Stevens-Johnson syndrome, which is characterized by a high mortality risk among patients receiving combination immunotherapy [10]. For targeted therapies, ePRO facilitated the management of rare toxicities, such as osimertinib-induced cutaneous vasculitis (case 3) [11], and provided an additional safety layer for outpatient monitoring of infusion-related reactions (case 4) [12]. These cases collectively illustrate how the EMA-EMI framework mitigates recall bias and enhances ecological validity by capturing subtle symptom changes in the patient’s natural environment. Furthermore, our results suggest that ePRO should not be viewed as a stand-alone monitoring tool but as a core component of a comprehensive PSP [4]. The integration of real-time data into clinical workflows supports a transition from passive compliance to an active concordance model, in which treatment decisions are made through shared decision-making based on the patient’s lived experience and real-time status [6,7]. Despite these promising results, several challenges remain. Adherence tended to decline during periods of symptom stability, suggesting that future iterations of the system could benefit from behavioral economics or gamification strategies to maintain long-term engagement [13]. Additionally, as 57.1% of our cohort was aged ≥70 years, family members often assisted with data entry. This introduces the potential for interrater variability, as proxies may overestimate symptom severity compared to patient self-reports [14]. This study has several limitations. First, as a single-center prospective cohort study with a relatively small sample size (N=35), the generalizability of our findings to broader populations may be limited. Second, the absence of a control group receiving conventional care makes it difficult to definitively determine whether the ePRO-EMI approach is superior to conventional care. Finally, although we used a descriptive thematic analysis to categorize patient feedback, the qualitative analysis was conducted on a limited dataset from a single-center cohort. Therefore, these findings should be interpreted as exploratory and illustrative of this specific clinical setting, rather than as fully generalizable to all older adults with cancer. In addition, the long-term sustainability and scalability of digital health interventions remain challenging, particularly in aging populations with heterogeneous levels of digital literacy and varying support systems [15]. In conclusion, this study provides real-world evidence that ePRO-based monitoring is feasible and beneficial for older patients with lung cancer. Future research should integrate artificial intelligence to provide automated, predictive warnings of toxicities and to foster deeper collaboration with regional health care systems [16]. Ultimately, digital transformation in health care, centered on patient-reported data, holds the potential to significantly improve patient well-being and safety in an aging society.

### Conclusions

ePRO integrated with EMA and EMI is feasible and clinically valuable for older patients with lung cancer in real-world settings. Real-time monitoring enabled the early detection of clinically significant adverse events and supported patient-centered care beyond routine outpatient visits. However, successful implementation requires continuous institutional support, improved digital literacy, and integration into comprehensive PSPs. Future research should focus on artificial intelligence–assisted toxicity prediction and broader regional health care collaboration to optimize sustainable implementation in aging societies.

## Supplementary material

10.2196/97890 Multimedia Appendix 1 Overview of the electronic patient-reported outcome system (Welby My Carte ONC).

10.2196/97890 Multimedia Appendix 2 Ecological momentary assessment–ecological momentary intervention workflow and clinical response framework.

10.2196/97890 Multimedia Appendix 3 Questionnaire items for patient evaluation.

10.2196/97890 Multimedia Appendix 4 STROBE (Strengthening the Reporting of Observational Studies in Epidemiology) checklist for cohort studies.

## Abbreviations

EMA: ecological momentary assessment
EMI: ecological momentary intervention
ePRO: electronic patient-reported outcome
ICI: immune checkpoint inhibitor
PS: performance status
PSP: patient support program
STROBE: Strengthening the Reporting of Observational Studies in Epidemiology
TKI: tyrosine kinase inhibitor
