# Supplementary figures and images for: Real-World Impact of Electronic Patient-Reported Outcomes on Early Intervention Among Older Patients With Lung Cancer: Prospective Cohort Study
Source: JMIR Cancer. 2026 Jul 6;12:e97890. doi: 10.2196/97890 (PMC13335941; doi:10.2196/97890)

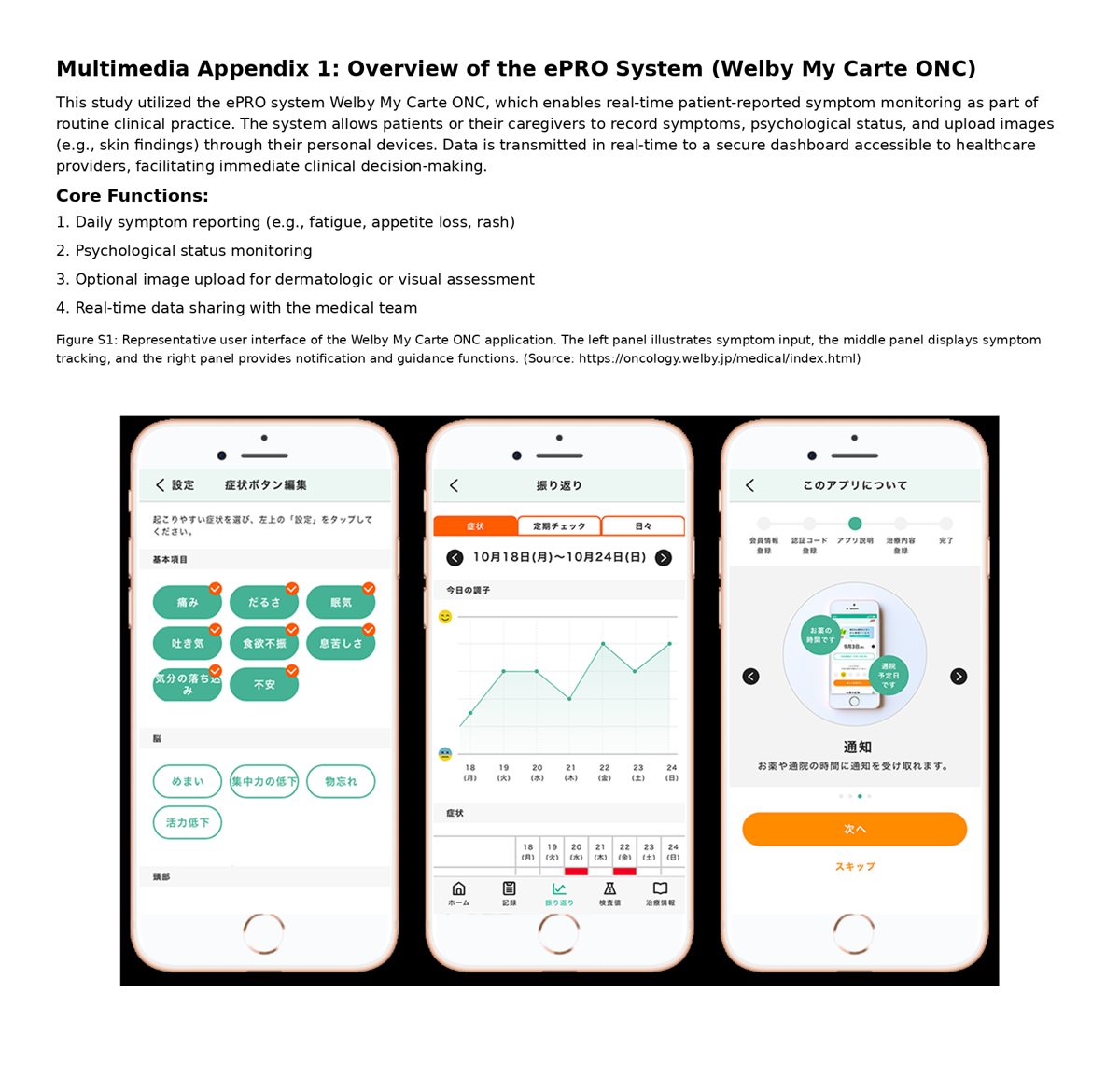

Supplement: Multimedia Appendix 1 [file cancer-v12-e97890-s001.png]

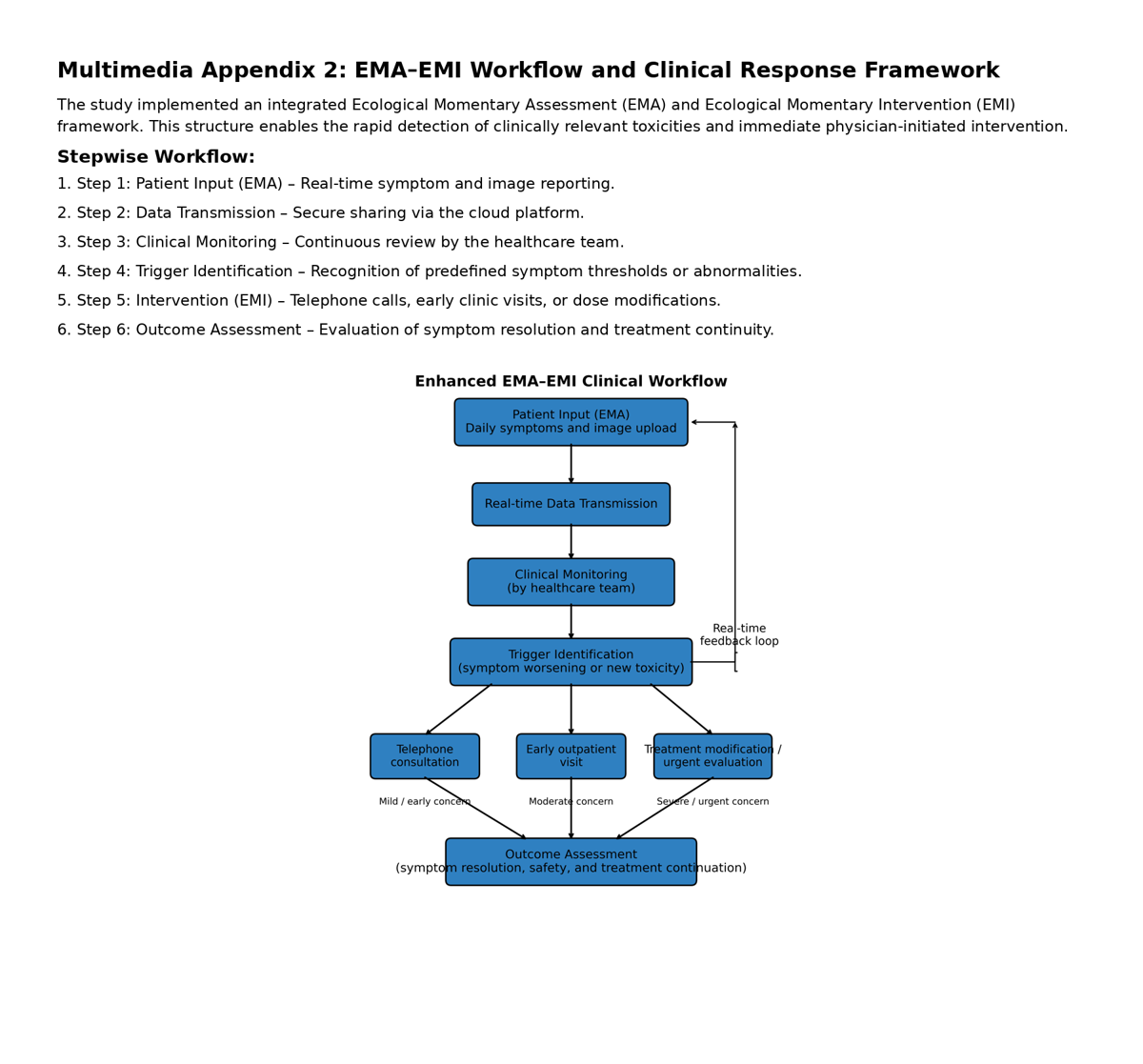

Supplement: Multimedia Appendix 2 [file cancer-v12-e97890-s002.png]

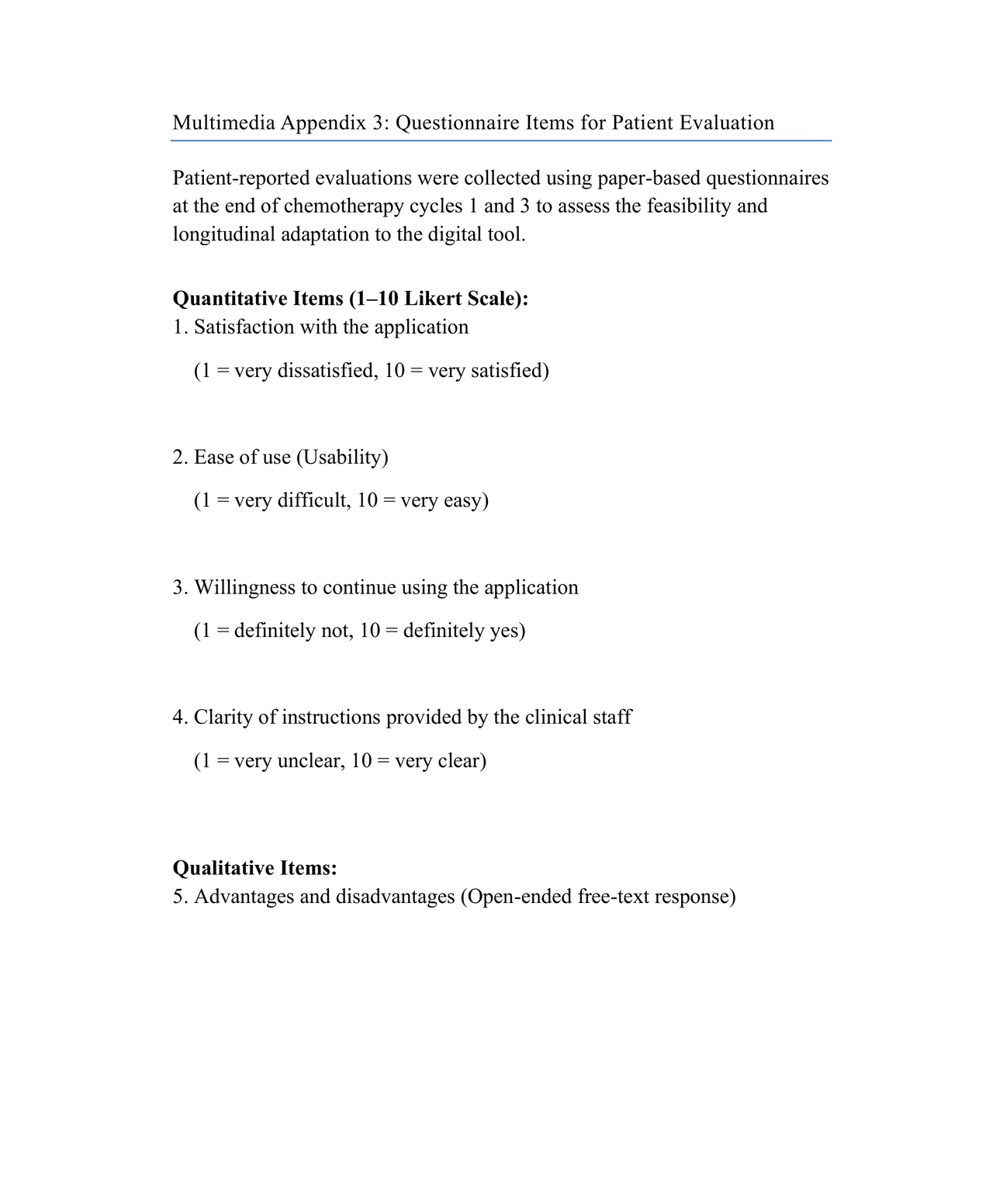

Supplement: Multimedia Appendix 3 [file cancer-v12-e97890-s003.png]

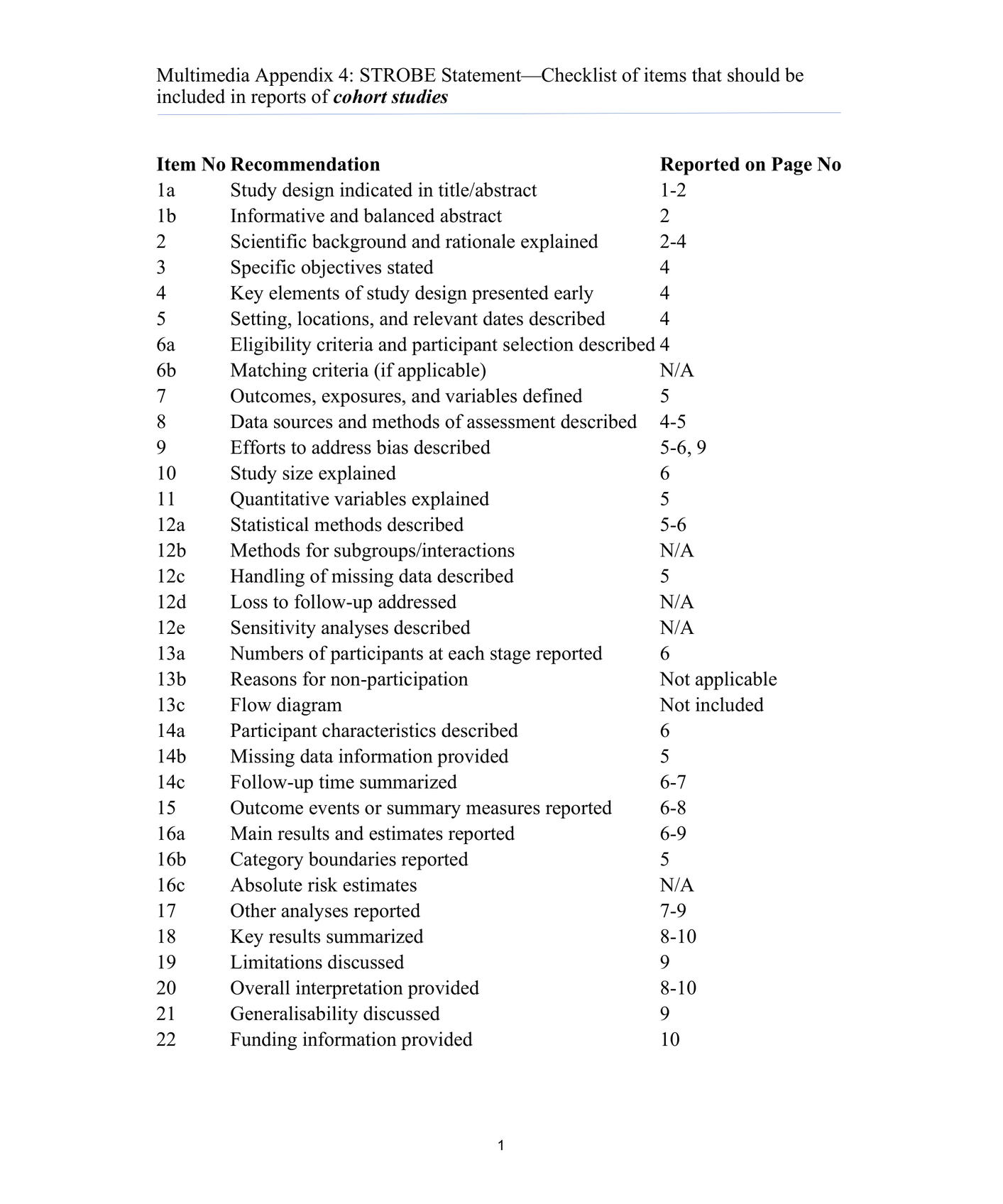

Supplement: Multimedia Appendix 4 [file cancer-v12-e97890-s004.png]
